# Large Differences in the Haptophyte *Phaeocystis globosa* Mitochondrial Genomes Driven by Repeat Amplifications

**DOI:** 10.3389/fmicb.2021.676447

**Published:** 2021-07-02

**Authors:** Huiyin Song, Yang Chen, Feng Liu, Nansheng Chen

**Affiliations:** ^1^CAS Key Laboratory of Marine Ecology and Environmental Sciences, Institute of Oceanology, Chinese Academy of Sciences, Qingdao, China; ^2^Laboratory of Marine Ecology and Environmental Science, Qingdao National Laboratory for Marine Science and Technology, Qingdao, China; ^3^Center for Ocean Mega-Science, Chinese Academy of Sciences, Qingdao, China; ^4^School of Earth and Planetary, University of Chinese Academy of Sciences, Beijing, China; ^5^Department of Molecular Biology and Biochemistry, Simon Fraser University, Burnaby, BC, Canada

**Keywords:** mtDNA sizes, mitochondrial genomes, *Phaeocystis globosa*, repeat region, Haptophyta

## Abstract

The haptophyte *Phaeocystis globosa* is a well-known species for its pivotal role in global carbon and sulfur cycles and for its capability of forming harmful algal blooms (HABs) with serious ecological consequences. Its mitochondrial genome (mtDNA) sequence has been reported in 2014 but it remains incomplete due to its long repeat sequences. In this study, we constructed the first full-length mtDNA of *P. globosa*, which was a circular genome with a size of 43,585 bp by applying the PacBio single molecular sequencing method. The mtDNA of this *P. globosa* strain (CNS00066), which was isolated from the Beibu Gulf, China, encoded 19 protein-coding genes (PCGs), 25 tRNA genes, and two rRNA genes. It contained two large repeat regions of 6.7 kb and ∼14.0 kb in length, respectively. The combined length of these two repeat regions, which were missing from the previous mtDNA assembly, accounted for almost half of the entire mtDNA and represented the longest repeat region among all sequenced haptophyte mtDNAs. In this study, we tested the hypothesis that repeat unit amplification is a driving force for different mtDNA sizes. Comparative analysis of mtDNAs of five additional *P. globosa* strains (four strains obtained in this study, and one strain previously published) revealed that all six mtDNAs shared identical numbers of genes but with dramatically different repeat regions. A homologous repeat unit was identified but with hugely different numbers of copies in all *P. globosa* strains. Thus, repeat amplification may represent an important driving force of mtDNA evolution in *P. globosa*.

## Introduction

The haptophyte *Phaeocystis globosa* is a cosmopolitan phytoplankton that plays a pivotal role in global carbon and sulfur cycles by releasing substantial quantities of dimethylsulfide propionate (DMSP) (Schoemann et al., 2005). It is also an important HAB species that causes blooms in various ocean regions including the Arabian sea, the Southern North Sea, and the coastal waters of East Asia and Southeast Asia (Madhupratap et al., 2000; Chen et al., 2002; Schoemann et al., 2005; Hai et al., 2010; Rousseau et al., 2013; Shen and Qi, 2021). *P. globosa* blooms outbreak frequently, bringing serious ecological hazards, and causing great economic losses of aquaculture industry and nuclear power facility threats (Shen et al., 2000; Qi et al., 2004; Hai et al., 2010; Cao et al., 2017).

Accumulating evidence suggested the *P. globosa* has high level of genetic diversity. Various physiological and ecological characteristics were observed among different *P. globosa* strains. For example, *P. globosa* strains collected in different ocean regions (e.g., Hong Kong and Shantou, China) have different acute toxic effects to *Artemia sinica* (Wei and Jiang, 2005). Sizes of *P. globosa* colonies found in the Chinese coastal waters can reach 3 cm in diameters, which are substantially larger than those found in European waters (Qi et al., 2004; Rousseau et al., 2007). The temperatures at which *P. globosa* blooms occurred were different for *P. globosa* blooms recorded in different geographical regions. For example, most *P. globosa* blooms occurred in Chinese coastal waters when temperatures were 16–22°C (Shen et al., 2000; Qi et al., 2004), which were higher than that in European waters (Seuront et al., 2006; Blauw et al., 2010). Cell surface structures of *P. globosa* strains isolated in Europe and Beibu Gulf, China were different. For example, small circular bulges were observed in small flagellate cells of China strains, while such characteristics were not observed in European strains (Hu et al., 2019).

Much effort has been made to ascertain *P. globosa* genetic diversity as a way to understand phenotypical differences. Common molecular markers (18S rDNA, 28S rDNA, ITS, and *rbcL*) have been applied to distinguish *P. globosa* strains with limited success (Hu et al., 2019; Song et al., 2020). The ITS region has been demonstrated to be a poor molecular marker because it has been found to have high intra-genome variation, while 18S rDNA, 28S rDNA and *rbcL* have been found to be over conservative that they cannot be used to effectively distinguish different strains (Hu et al., 2019; Song et al., 2020). Thus, these common molecular markers could not be adequately used to probe *P. globosa* genetic or phenotypic diversity. Molecular markers with high resolution are urgently needed to resolve *P. globosa* genetic diversity in environment.

Comparative analysis of the organelle genomes has been fruitful for identifying molecular markers with improved resolution. The complete chloroplast genome (cpDNA) of the first *P. globosa* strain was published in 2014, which was 107,461 bp in length (Smith et al., 2014). One molecular marker *rbcS-rpl*27 based chloroplast sequence intergenic spacer was found to be able to separate phylogeographic populations of *P. globosa* (Zhang et al., 2021). Comparative analysis of the cpDNAs of *P. globosa* strains facilitated the development of a new molecular marker *pgcp1* with even higher resolution for tracking *P. globosa* genetic diversity (Song et al., 2020).

Relative mutation rate of mtDNAs has been found to be higher than that of cpDNAs in *P. globosa* (Smith et al., 2014). The mtDNAs of *P. globosa* and *P. antarctica* have been published but they are still incomplete, due to the complex repeat regions (Smith et al., 2014). Repeat regions are ubiquitous in algal mtDNAs, some of which can be very large in length. For example, mtDNA of the cryptophyte *Rhodomonas salina* contains a 4.7 kb long repeat region, 11 repeat motifs ∼40–700 bp long occurring up to 31 times, forming a complex repeat structure. Tandem repeats are the major arrangement but the region also includes a large, 3 kb, inverted repeat and several potentially stable 40–80 bp long hairpin structures (Hauth et al., 2005). The cryptophyte *Hemiselmis andersonii* has a 20 kb repeat region, comprised of numerous tandem and dispersed repeat units of between 22 and 336 bp, and occurring up to 100 times. Adjacent to these repeats are 27 copies of palindromic sequences predicted to form stable DNA stem-loop structures (Kim et al., 2008). The mtDNA of *Phaeodactylum tricornutum* has an even larger repeat region of 35 kb, consisting almost entirely of various combinations of several kinds of tandem repeat, including five elements 33-404 bp (Oudot-Le Secq and Green, 2011). The chlorophyte *Pedinomonas* mtDNA contains a 9 kb-repeated sequence, 13 repeat elements range in size from 6 to 389 bp (*ele-01* to *ele-13*) were characterized. Although most members within an element family are identical, some are not (Turmel et al., 1999). We hypothesize that change in repeat regions may be, at least partially, due to differential repeat unit amplification, which is an important driving force for *P. globosa* mtDNA differences in evolution.

In this study, we constructed the first complete mtDNA of *P. globosa*, which contained two large repeat regions of 6.7 kb and ∼14.0 kb in length, respectively. We identified several repeat units with various numbers of copies. Comparative analysis of six *P. globosa* mtDNAs revealed that the copies numbers varied substantially. Our results revealed that change in repeat units is a driver of *P. globosa* mtDNA diversity.

## Materials and Methods

### *Phaeocystis globosa* Strain Isolation, Culture and Characterization

The five *P. globosa* strains (CNS00066, CNS00069, CNS00087, CNS00262, and CNS00266) described herein were isolated from samples collected in 2019 from *P. globosa* bloom waters of the Beibu Gulf, China and Vietnam sea area (Table 1). Single-cell isolation was achieved by selecting individual cells using a micropipette, followed by multiple washes before transferring each single cell to 24-well culture dishes for growth and characterization. They grew in L1 seawater culture medium (without Na_2_SiO_3_) (Guillard and Hargreaves, 1993). Cultures were maintained at 20°C under a 12:12 h light: dark cycle with approximately 30 μM s^–1^ m^–2^ of cool white fluorescent illumination.

**TABLE 1:** *Phaeocystis globosa* strains information used in this study.

strains	Geographic origin	18S rDNA Accession number	Sampling time	Longitude (E°)	Latitude (N°)
CNS00066	Beibu Gulf, China	MN927483	2019.02	109.00	20.50
CNS00069	Beibu Gulf, China	MN927485	2019.02	109.00	21.17
CNS00087	Vietnam sea area	MN927500	2019.08	108.13	10.60
CNS00262	Vietnam sea area	MW575291	2019.08	108.13	10.60
CNS00266	Vietnam sea area	MW575290	2019.08	108.13	10.60

Identification of the cultured algae was done based on microscopic morphological characters and phylogenetic analysis based on 18S rDNA. ZEISS Axio Imager Z2 light microscope (Zeiss, Sliedrecht, Netherlands) were used for morphological observation. 18S rDNA was assembled basing Illumina reads with GetOrganelle (Jin et al., 2020), with publicly available 18S rDNA sequences of *P. globosa* as reference sequences. The assembled sequences were further validated by aligning sequencing reads against the assembled result using BWA (Li and Durbin, 2009) and SAMtools (1.9) (Li et al., 2009) and visualized using IGV (Thorvaldsdottir et al., 2013). For phylogenetic analysis, 40 18S rDNA sequences were used. The sequences were aligned using MAFFT (Katoh and Standley, 2013). The ambiguously aligned regions were further manually edited and adjusted using MEGA7.0 (Kumar et al., 2016). There were a total of 1562 positions in the final dataset. The maximum likelihood (ML) tree and aBayes (BI) tree based on 18S rDNA were inferred using W-IQ-TREE, with best substitution model TNe + I + G4 was selected using default parameters of W-IQ-TREE (Trifinopoulos et al., 2016). The support for nodes was assessed by performing 1000 bootstrap replicates. For analysis of sequence variability between *Phaeocystis* strains, 1633 positions in the final 18S rDNA sequence matrix, 420 positions in the 18S rDNA V4 sequence matrix were obtained, 171 positions in the 18S rDNA V9 sequence matrix were obtained. Numbers of pair-wise variations sites among *P. globosa* strains were calculated using BioEdit (Hall, 1999).

### DNA Preparation and Genome Shotgun Sequencing

Cultures at the exponential growth phase were harvested and concentrated by centrifugation. Two sequencing methods were used for the genomes in this study. All five strains obtained in this study were sequenced using Illumina sequencing, while the strain CNS00066 was also sequenced using PacBio Sequel II sequencing technology.

For Illumina sequencing, total nucleic acids were extracted using the OMEGA HP Plant DNA Mini Kit (Omega Bio-tek, Inc., United States) and quantified using a NanoDrop One spectrophotometer (Labtech International Ltd., Uckfield, United Kingdom). DNA samples of five *P. globosa* strains were prepared for whole genome sequencing. DNA degradation and contamination were monitored on 1% agarose gels. DNA concentration was measured using Qubit^®^ DNA Assay Kit in Qubit^®^ 2.0 Flurometer (Life Technologies, CA, United States). Sequencing libraries were generated using NEBNext^®^ DNA Library Prep Kit following manufacturer’s recommendations and indices were added to each sample. The genomic DNA was randomly fragmented to a size of 350 bp by shearing, then DNA fragments were end polished, A-tailed, and ligated with the NEBNext adapter for Illumina sequencing, and further PCR enriched by generic adapter P5 and P7 oligos. The PCR products were purified (AMPure XP system) and resulted in libraries were analyzed for size distribution by Agilent 2100 Bioanalyzer system and quantified using real-time PCR. Qualified libraries were sequenced on an Illumina platform according to effective concentration and data volume at Novogene (Beijing, China). Sequencing was done using NovaSeq PE150 (Illumina, San Diego, CA, United States).

For strain CNS00066, the long-read DNA sequencing technology PacBio Sequel II was used for constructed the complete mitochondrion genome. For PacBio Sequel II sequencing, high quality genomic DNA was extracted using a modified CTAB method. RNase A was used to remove RNA contaminants. The quality of the DNA was checked by agarose gel electrophoresis. One long insert library (15 kb) was prepared, which was sequenced using the PacBio Sequel II platform (Frasergen, Wuhan, China). We generated 6.44 Gb HiFi reads.

### Quality Control and Assembly

For Illumina sequencing data, methods of quality control and assembly were similar with Song et al. (2020). Raw reads in FASTQ format were first processed through a series of quality control (QC) procedures with in-house C scripts by (1) removing reads with ≥ 10% unidentified nucleotides (N); (2) removing reads with > 50% bases having Phred quality < 5; (3) removing reads with > 10 bp aligned to the adapters, allowing ≤ 10% mismatches; (4) removing putative PCR duplicates generated by PCR amplification in the library construction process (read 1 and read 2 of two paired-end reads that were completely identical). The filtered reads were assembled with SPAdes (Bankevich et al., 2012) and GetOrganelle (Jin et al., 2020).

The resulting assembly scaffolds from the mtDNA were determined using BLAST (Johnson et al., 2008) of the complete mtDNA of the strain CNS00066 and publicly available partial mtDNA of *P. globosa* strain Pg-G(A) (NC_021637) (Smith et al., 2014). One mtDNA scaffold which contained one N region was obtained for each strain, respectively. Finally, clean reads were mapped to the assembled mtDNA to validate and to correct the wrong bases, using BWA (Li and Durbin, 2009) and SAMtools (1.9) (Li et al., 2009) and visualized using IGV (Thorvaldsdottir et al., 2013). We successfully completed the repeat region 1 in the strains CNS00069, CNS00087, CNS00262, and CNS00266, all of which were validated using Sanger DNA sequencing. The sequences were further validated by aligning sequencing reads against the assembled sequences using BWA (Li and Durbin, 2009) and SAMtools (1.9) (Li et al., 2009) and visualized using IGV (Thorvaldsdottir et al., 2013).

For PacBio Sequel II sequencing data, data were mapped to partial *P. globosa* mtDNA (KC967226) with minimap2 (version 2.5) (Li et al., 2018) using the default parameters, and a total 7.03Mb of potential mitochondrial HiFi reads were obtained. Reads depth of coverage was > 160×. The mtDNA HiFi reads were locally assembled with MECAT2 software (default parameter) (Xiao et al., 2017) to obtain circular mitochondrial contig 43,585 bp. The mtDNA was validated through an additional round of alignment using BWA (Li and Durbin, 2009) and SAMtools (1.9) (Li et al., 2009) and visualized using IGV (Thorvaldsdottir et al., 2013).

In order to facilitate comparative analysis, the starting sites of mtDNAs were all adjusted to *cox*1.

### Annotation of *P. globosa* mtDNA and Phylogenetic Analysis of Haptophyta

The mtDNAs was annotated with GeSeq (Tillich et al., 2017), ORF-FINDER (Rombel et al., 2002), and BLAST^1^ homology searches. Transfer RNAs (tRNAs) and ribosomal RNAs (rRNAs) were identified using tRNAscan-SE 2.0 (Lowe and Chan, 2016) and RNAmmer (Lagesen et al., 2007), respectively. The annotated data were deposited into NCBI GenBank database under the accession number of MW435860-MW435863, and MW435865. The graphical gene map was designed with the OrganellarGenomeDRAW program^2^ (Greiner et al., 2019). The phylogenetic tree of Haptophyta was constructed and the analysis involved 18 haptophyte taxa with two cryptophyte members as an outgroup. The dataset was composed of 3959 sites and assembled from the following 13 proteins: ATP6, ATP9, COX1, COX2, COX3, COB, NAD1, NAD2, NAD3, NAD4L, NAD4, NAD5, and NAD6. All amino acid sequences were aligned using MAFFT (Katoh and Standley, 2013). Regions showing poor alignment were trimmed with trimAl v1.2 (Capella-Gutierrez et al., 2009) using the parameter -automated1, and total amino acid sequence were concatenated using ALTER (Glez-Pena et al., 2010). There was a total of 3959 positions in the final dataset. Maximum likelihood (ML) tree and aBayes (BI) tree were inferred using W-IQ-TREE. The dataset was partitioned by protein, and best substitution model of each partition was estimated using default parameters of W-IQ-TREE (Trifinopoulos et al., 2016), the mtZOA + G4 substitution model for ATP6, COX2, COX3,COB, NAD1, NAD3, NAD4l proteins, mtZOA + I substitution model for ATP9 protein, mtZOA + I + G4 substitution model for COX1 protein, mtInv + F + G4 substitution model for NAD2 protein, mtZOA + F + G4 substitution model for NAD4 protein, mtZOA + F + I + G4 substitution model for NAD5 protein, cpREV + F + I + G4 substitution model for NAD6 protein. Bootstrap analysis was performed using the ultrafast bootstrap approximation with 1000 replicates.

### Synteny Analysis

The synteny comparison of seven mtDNAs of *Phaeocystis* was visualized using Mauve ver. 2.3.1 under the progressive mode (Darling et al., 2010). The gene content and synteny of strain CNS00069, strain Pg-G(A) and *P. antarctica* strain CCMP1374 compared to that of strain CNS00066, respectively, was performed using circos-0.69 (Krzywinski et al., 2009).

### Repeat Analysis

A dot-plot similarity matrix of *P. globosa* strain CNS00066 mtDNA against itself was done with Dotter (Barson and Griffiths, 2016), in order to show repeat regions. The repeat regions were analyzed using Tandem Repeats Finder (Benson, 1999) and local BLAST using this mtDNA as the database. For stem–loop structure prediction and calculation of minimum Gibbs free energies in DNA, we utilized RNAstructure^3^ used default parameters (Bellaousov et al., 2013). Here, we considered only stem–loop structures which were max expect results predicted, although numerous smaller and less stable structures. The repeat units sequence matrix was aligned by MAFFT (Katoh and Standley, 2013), and the ambiguously aligned regions were further manually edited and adjusted by eye. The sequence similarity was analyzed using BioEdit (Hall, 1999).

## Results

### Structural Features of the mtDNAs and Base Composition

**FIGURE 1:**
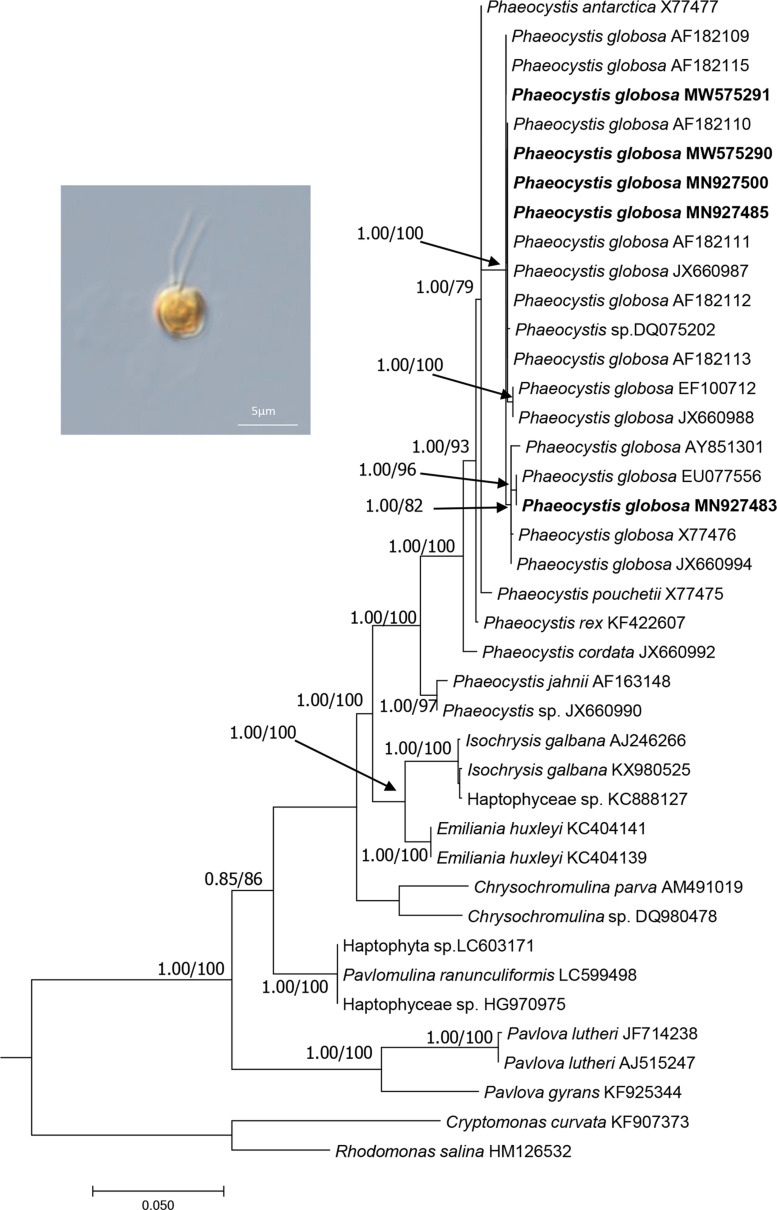
The phylogenetic analysis of the haptophyte species based on 18S rDNA sequences. The evolutionary history was inferred by using the Maximum Likelihood method and aBayes (BI) method based on TNe + I + G4 substitution model. The analysis involved 40 18S rDNA sequences. There were a total of 1562 positions in the final dataset. Evolutionary analyses were conducted using W-IQ-TREE. Numbers at the branches represent aBayes support/ultrafast bootstrap support (%), respectively. Only bootstrap values above 0.50 aBayes support and 50% ML bootstrap support are shown. The strains obtained in this study were bold. Morphology of strain CNS00066 was shown.

Five *P. globosa* strains (CNS00066, CNS00069, CNS00087, CNS00262, and CNS00266) obtained in this study were identified based on comparative analysis of 18S rDNA sequences. The five *P. globosa* strains were nested within *P. globosa* monophyletic clade with robust support (Figure 1). The numbers of nucleotide differences between *P. globosa* strains were 0-16 nucleotides, which were much smaller than that of other congeneric species (19-74 nucleotides). V4 region of 18S rDNA was also analyzed, and the numbers of nucleotide differences between *P. globosa* strains were 0-6 nucleotides, which were much smaller than that of other congeneric species (11-20 nucleotides). 18 of 25 *Phaeocystis* 18S rDNA sequences have complete V9 region, and we analyzed the V9 region of these 18 strains. The number of V9 region nucleotide differences between *P. globosa* strains were 0-2 nucleotides, which was indistinguishable from other congeneric species (0-20 nucleotides). This indicated that V9 region does not provide enough resolution for species identification in *Phaeocystis*.

Of these five *P. globosa* strains, CNS00066 was a solitary-celled (Figure 1), which never formed colonies under laboratory culture condition, although it was originally isolated from a *P. globosa* colony. CNS00066 cells were 3-4.5 μm in diameter with two flagella and one haptonema emerging from a depression in the cell body. Strains CNS00069, CNS00087, CNS00262, and CNS00266 had both solitary cells and colonies, with cells being 4-8 μm in diameter, and colonies being up to 0.3 cm in diameter. Some cells in colonies had flagella and haptonema, while other did not.

**FIGURE 2:**
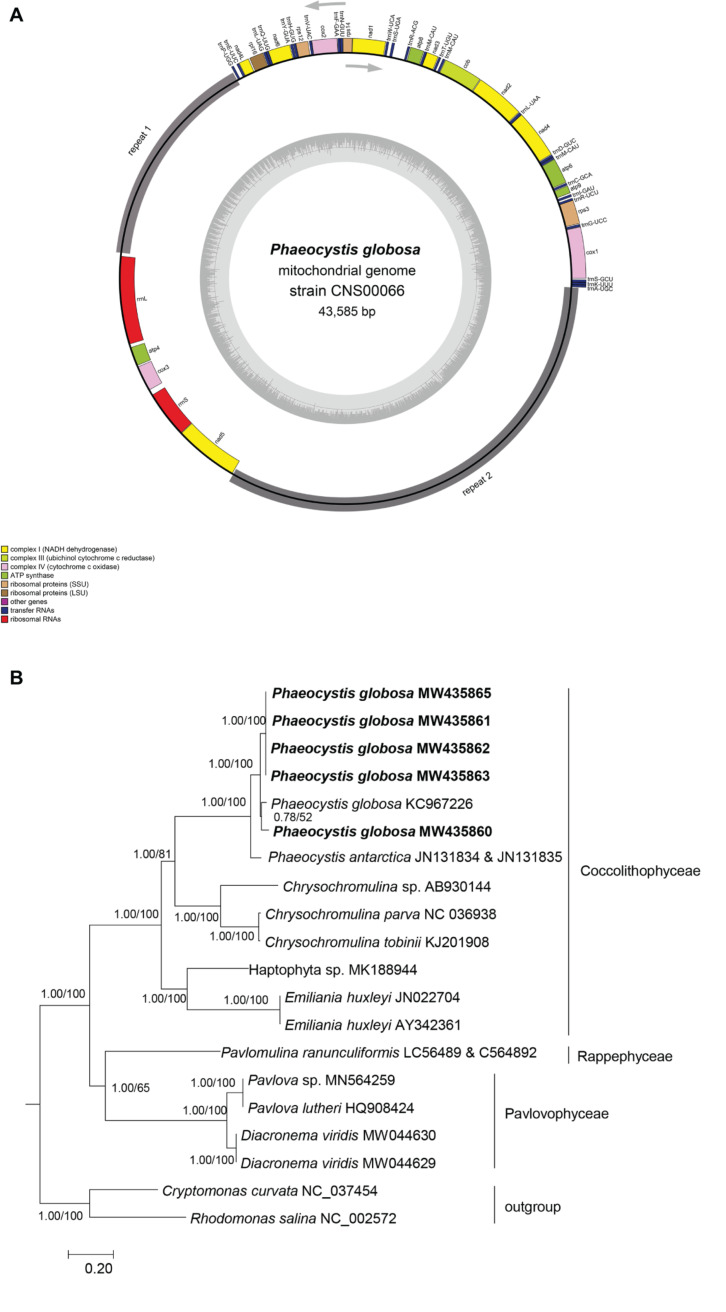
Circular map of the *P. globosa* mtDNA and phylogenetic analysis of haptophyte species. **(A)** Circular map of the mtDNA of *P. globosa* strain CNS00066. Genes were colored according to the functional categories listed in the index below the map. The GC content is indicated on the inner circle. The repeat regions (repeat 1 and repeat 2) is in gray. Genes showed inside and outside the circle are transcribed clockwise and counter-clockwise, respectively. **(B)** Phylogenetic tree of haptophyte species based on 13 mitochondrial proteins (ATP6, ATP9, COX1, COX2, COX3, COB, NAD1, NAD2, NAD3, NAD4L, NAD4, NAD5, and NAD6). Phylogenetic tree was reconstructed on the IQ-TREE web server (Trifinopoulos et al., 2016). Numbers at the branches represent aBayes support/ultrafast bootstrap support (%), respectively. Our strains were shown in bold.

The mtDNA of *P. globosa* strain CNS00066 was a circular molecule of 43,585 bp (Figure 2A). It had two large blocks of repeat regions. The repeat regions segregated the coding region into two parts and the genes were encoded on two strands, respectively, suggesting that the genome may be transcribed in two units. Aside from the repeat region, the mtDNA was relatively compact with 92.8% of sequence specifying genes and structural RNAs, with only 7.2% non-coding regions. The overall AT content of the mtDNA of CNS00066 was 70.6%, which was similar to mtDNAs of other species in the Coccolithophyceae (also known as Prymnesiophyceae, consisting of Phaeocystales, Isochrysidales, and Prymnesiales) (66.7-71.7%), which was higher than that of species in Pavlovophyceae (Pavlovales) (60.8-62.7%) (Table 2). The longest intergenic spacer was located between *trnR-ACG* and *trnS-UGA*, which was 379 bp.

**TABLE 2:** The known mitochondrial genomes from 19 taxa in the haptophyte members.

Class	Order	Species	Accession number	The known repeat length (>250 bp)	Size (bp)	A + T (%)	References
Coccolithophyceae	Phaeocystales	*Phaeocystis globosa* CNS00066	MW435860	two, 6.7 kb + 14 kb	43,585	70.6	This study
		*Phaeocystis globosa* CNS00069	MW435863	∼343 bp	24,330 (partial)	66.7	This study
		*Phaeocystis globosa* CNS00087	MW435865	∼1.4 kb	25,390 (partial)	67.2	This study
		*Phaeocystis globosa* CNS00262	MW435861	∼705 bp	24,764 (partial)	66.9	This study
		*Phaeocystis globosa* CNS00266	MW435862	∼334 bp	24,328 (partial)	66.7	This study
		*Phaeocystis globosa* Pg-G(A)	KC967226	∼709 bp	24,477 (partial)	69.5	Smith et al., 2014
		*Phaeocystis antarctica* CCMP1374	JN131834 & JN131835	∼763 bp	19,177 + 8,370 (partial)	70.3	Smith et al., 2014
	Isochrysidales	*Emiliania huxleyi* CCMP1516	JN022704	∼1.9 kb	28660	71.5	Smith and Keeling, 2012
		*Emiliania huxleyi* CCMP 373	AY342361	∼2.2 kb	29,013	71.7	Puerta et al., 2004
	Prymnesiales	*Chrysochromulina tobinii* CCMP291	KJ201908	∼9.3 kb	34,288	68.6	Hovde et al., 2014
		*Chrysochromulina parva* UW 1161	NC_036938	none	24,009 (partial)	67.5	Hovde et al., 2019
		*Chrysochromulina* sp. NIES-1333	AB930144	two, ∼1.6 kb + 1.6 kb	34,291	70.0	Nishimura et al., 2014
	Incertae sedis	Haptophyta sp. isolate H2	MK188944	none	25,180	77.9	Wideman et al., 2020
Pavlovophyceae	Pavlovales	*Pavlova lutheri* CCMP 1325	HQ908424	four, ∼6 kb	34,086 (partial)	62.7	–
		*Pavlova* sp. NIVA-4/92	MN564259	three, ∼7.3 kb	36,202	62.5	Hulatt et al., 2020
		*Diacronema viridis* voucher KMMCC 0113	MW044630	∼3.8 kb	29,282	60.8	–
		*Diacronema viridis* culture CCMP:620	MW044629	∼3.8 kb	29,282	60.8	–
Rappephyceae	Pavlomulinales	*Pavlomulina ranunculiformis* NIES-3900	LC564891 & LC564892	none	11,199 + 9,863 (partial)	54.1	Kawachi et al., 2021

*“–” represent no references.*

### Phylogenetic Analysis of Haptophyte Species

To explore the evolutionary relationship between *P. globosa* and other haptophyte members, we constructed a phylogenetic tree using protein sequences encoded by 13 PGCs in the mtDNAs that were shared by all haptophyte members (Figure 2B). The mtDNAs of five strains obtained in this study clustered with other *P. globosa* strains in a monophyletic clade with robust support. The mtDNAs of strain CNS00066 (MW435860) clustered with *P. globosa* (KC967226) with moderate support. The mtDNAs of four strains CNS00069 (MW435863), CNS00087 (MW435865), CNS00262 (MW435861), and CNS00266 (MW435862), which were isolated from the Beibu Gulf in China and Vietnam sea area, clustered together in a different clade. The species in Coccolithophyceae, Pavlovophyceae, and Rappephyceae all formed well supported branches, and Rappephyceae was sister to the Pavlovophyceae. The phylogenetic relationship was generally agreed with the 18S rDNA-based phylogenetic relationship in this study and previous results basing mitochondrial and plastid dataset (Kawachi et al., 2021) with minor differences, which Rappephyceae was sister to the Coccolithophyceae.

### Gene Content and Synteny Analysis of *Phaeocystis* Species

**FIGURE 3:**
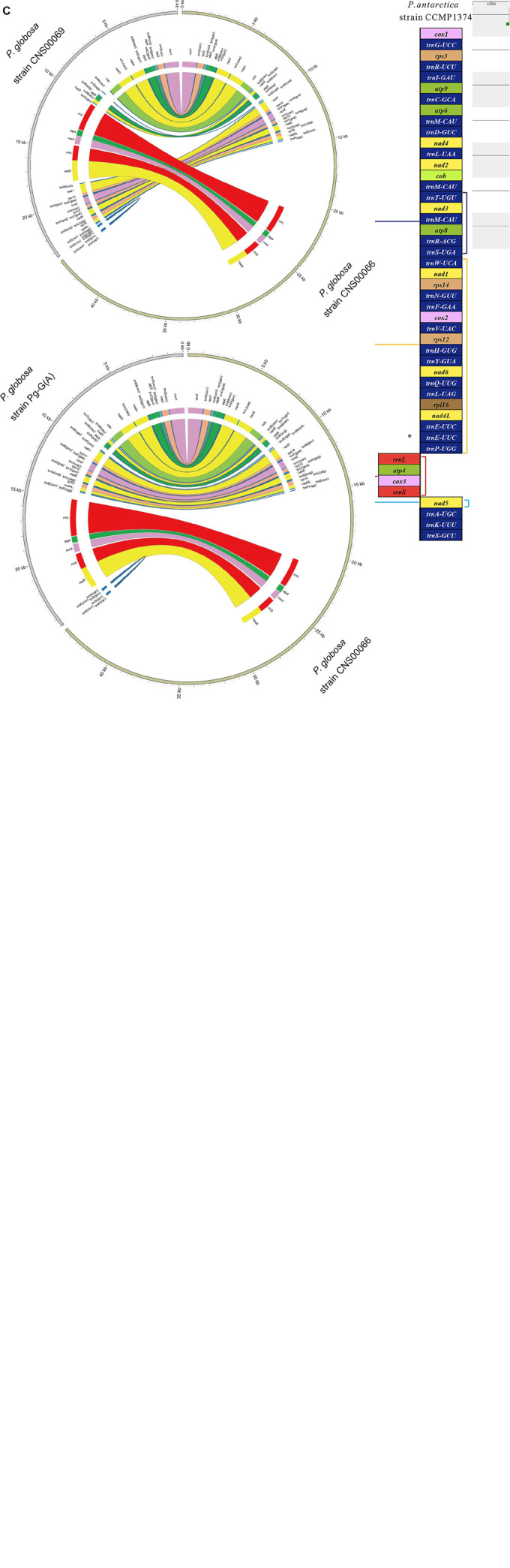
Multicollinearity of seven mtDNAs of genus *Phaeocystis* members. **(A)** The genomes were aligned using Mauve 2.3.1. Each colored block indicates a region of synteny among the seven genomes. The block a contain *cox*1-*trnG-UCC-rps*3*-trnR-UCU-trnI-GAU-atp*9*-trnC-GCA-atp*6*-trnM-CAU-trnD-GUC-nad*4 *-trnL-UAA-nad*2*-cob-trnM-CAU* gene cluster, block b contain *trnT-UGU-nad*3*-trnM-CAU-atp*8*-trnR-ACG-trnS-UGA* gene cluster, and the block c contain *trnW-UCA-nad1-rps*14*-trnN-GUU-trnF-GAA-cox*2*-trnV-UAC-rps*12*-trnH-GUG-trnY-GUA-nad*6*-trnQ-UUG-trnL-UAG-rpl*16*-nad*4L*-trnE-UUC-trnP-UGG* gene cluster, and the block d contain *rrnL-atp*4*-cox*3*-rrnS* gene cluster, and the block e contain *nad*5 gene. **(B)** Comparison of genome organization and gene order among the genomes. The square brackets indicated the region of synteny among the genomes. Lines connecting blocks indicated putative homology. Compared with *P. globosa*, *P. antarctica* have the additional tRNA gene *trnE-UUC*, which is labeled by an asterisk (*). **(C)** The mitochondrion genomes content and synteny of strain CNS00069 and strain Pg-G(A) compared to that of strain CNS00066, respectively.

The six mtDNAs of *P. globosa* (five obtained in this study and KC967226) shared an identical number (19) of PCGs (Figures 3A-C). Four of these genes (*atp4*, *atp6*, *atp8* and *atp9*), which encode subunits of the ATP synthase complex, were identified. Seven genes encoding NADH dehydrogenase subunits, *nad1-6* and *nad4L*, were detected. Three genes encoding complex IV (cytochrome c oxidase subunits), *cox1*, *cox2* and *cox3* were identified. Four genes encoding ribosomal proteins, *rpl16*, *rps12*, *rps14* and *rps3* were detected. One gene encoding complex III (cytochrome c reductase) *cob* was identified. A total of 25 tRNA genes and two rRNA genes (*rrnL* and *rrnS*) are present in the *P. globosa* mtDNA. 5S rRNA gene was not detected. None of the genes contained intron except *rrnL* (23S rRNA) gene in mtDNA of *P. globosa* strain Pg-G(A), which contained a single intron.

We performed a comparative analysis of seven mtDNAs of the genus *Phaeocystis*. Comparison of genome organization and gene order were shown in Figure 3A and Supplementary Figure 1. Five conserved gene blocks (blocks a-e) were identified among mtDNAs of *Phaeocystis*. Within *P. globosa* strains, two gene arrangement events were discovered. The first was mtDNAs of strain CNS00066 and strain Pg-G(A), which showed identical gene orders (blocks a-b-c-d-e), and the second were mtDNAs of strains CNS00069, CNS00087, CNS00262, and CNS00266, which showed another identical gene order, and blocks order was a-b-d-e-c. Compared with the first genome organization, the block b was inverted and reversed, and the block c and the blocks d-e were reciprocally translocated in the second genome organization. A high degree of syntenic conservation was identified between *P. globosa* strains CNS00066, Pg-G(A) and *P. antarctica*, except that block e (*nad*5*)* was inverted and reversed in *P. antarctica*. Compared to *P. globosa*, another additional tRNA gene *trnE-UUC* was found in *P. antarctica*.

### Comparative Analysis of mtDNAs

**FIGURE 4:**
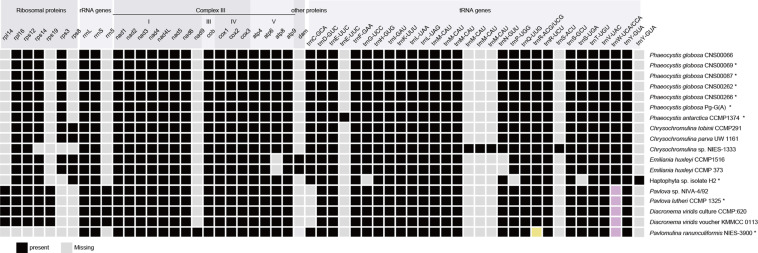
Comparison between mtDNA gene repertoires among haptophyte members. Black square, present; Light gray square, absent; Buff and pink represent distinctive anticodons. The asterisks indicate partial mtDNAs.

Comparison of 18 mtDNAs of species in the phylum Haptophyta (Phaeocystales, Isochrysidales, Prymnesiales, Pavlovales, and Pavlomulinales) revealed interesting features in gene content (Figure 4). They shared 36 core genes, including 14 PCGs *(atp4*, *atp6*, *atp9*, *cob*, *cox1*, *cox2*, *cox3*, *nad1*, *nad2*, *nad3*, *nad4*, *nad4L, nad5*, and *nad6*), two rRNAs (*rnl* and *rns*), and 20 tRNAs genes (*trnA-UGC*, *trnD-GUC*, *trnE-UUC*, *trnF-GAA*, *trnH-GUG*, *trnI-GAU*, *trnK-UUU*, *trnL-UAA*, *trnL-UAG*, *trnM-CAU*, *trnM-CAU*, *trnM-CAU*, *trnQ-UUG*, *trnR*, *trnR-UCU*, *trnS-GCU*, *trnT-UGU*, *trnV-UAC*, *trnW*, and *trnY-GUA*). These mtDNAs also showed important differences. For example, *rpl14* and *rps19* were found only in Pavlovales (Pavlovophyceae). The *rps14* was absent from *Chrysochromulina* sp. NIES-1333 (Prymnesiales) and *Pavlomulina ranunculiformis* NIES-3900, the *rps3* was absent from *Chrysochromulina* sp. NIES-1333 (Prymnesiales), Pavlovales, Haptophyta sp. isolate H2 and *P. ranunculiformis* NIES-3900. The *nad9* was found only in *P. ranunculiformis* NIES-3900. The *atp8* gene was absent only in Isochrysidales, while the *dam* was found only in the *Emiliania huxleyi* (Isochrysidales). The *dam* gene, which encodes a DNA adenine methyltransferase, is common in bacterial genomes. Using tblastn search, we found that *dam* gene was also present in a closely related species *Gephyrocapsa oceanica* (Isochrysidales) and distantly related *Aureococcus anophagefferens* (Ochrophyta) mtDNAs, but absent from other studied mtDNAs. It may be gained via horizontal gene transfer. Further investigation is needed to test this possibility. Compared with mtDNAs of other Haptophyte species, the *Chrysochromulina* sp. NIES-1333 mtDNA encoded three extra *trnM-CAU* genes and one extra *trnS-ACU* gene, leading to a total of six *trnM-CAU* genes and three *trnS* genes. The six *trnM-CAU* genes were dispersed in genome, with sequence identities ranging from 36.4% to 100%. In contrast, no obvious similarity was found among three *trnS* genes. The *rps8* gene was only found in Isochrysidales and Prymnesiales. *P. antarctica* (Phaeocystales) had an extra *trnE-UUC* gene. The 5S rRNA gene was found only in Pavlovales (Pavlovophyceae). The *trnG-UCC*, *trnP-UGG* and *trnS-UGA* genes were only absent in Haptophyta sp. isolate H2. The *trnW* gene contained UCA anticodon in Coccolithophyceae and CCA anticodon in Pavlovophyceae and Rappephyceae. The *trnR* gene contained ACG anticodon in Coccolithophyceae and Pavlovophyceae, a UCG anticodon in Rappephyceae.

Alernative start codons were used in different haptophyte mtDNAs. AUG was the primary start codon in haptophyte mtDNAs and only known start codon in mtDNAs of *Phaeocysits*. In addition, GUG may serve as the translation initiation codon for *atp4*, *rps8* in *E. huxleyi*, *atp9*, *nad4*, and *rpl14* in *D. viridis*, *atp4* in Haptophyta sp. isolate H2, *atp9*, *rpl14*, and *rps14* in *Pavlova*. The UUG may serve as the translation initiation codon for *atp6*, *atp9*, *nad4*, *nad4L*, *nad9* in *P. ranunculiformis* NIES-3900, *atp9*, *cox1*, *cox2*, *cox3*, *cob*, *nad1*, *nad4*, *rpl16*, and *rps3* in *Chrysochromulina* sp. NIES-1333, *cox3* in *C. tobinii* CCMP291, *cox2* and *nad2* in Haptophyta sp. isolate H2, and *rps14* in genus *Pavlova*.

**FIGURE 5:**
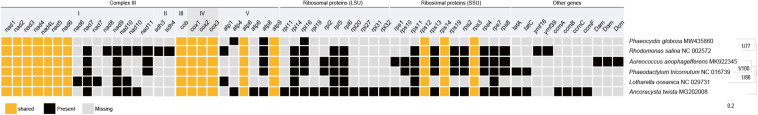
Comparison between mtDNA protein coding gene repertoires among Haptophyta (*P. globosa*) and other lineages mtDNAs. Black square, present; Light gray square, absent; Orange represents shared genes.

We also compared PCGs in mtDNAs of Haptophyte species with that of *Ancoracysta twista*, *Rhodomonas salina* (Cryptophyceae), *Aureococcus anophagefferens* (SAR clade, Pelagophyceae), *Phaeodactylum tricornutum* (SAR clade, Bacillariophyceae), *Lotharella oceanica* (SAR clade, Chlorarachniophyceae) (Figure 5). These six mtDNAs shared 16 genes, *atp6* and *atp9* genes encoded subunits of the ATP synthase complex, *cox1*, *cox2* and *cox3* genes encoded complex IV (cytochrome c oxidase subunits), *cob* gene encoded encoding complex III (cytochrome c reductase), *nad1*, *nad2*, *nad3*, *nad4L*, *nad4*, *nad5* and *nad6* genes encoded NADH dehydrogenase subunits. *rps3*, *rps12*, and *rps14* genes encoded ribosomal proteins. These genes are probably the most essentially important core genes and perform basic functions. Interestingly, *P globosa* mtDNA contained the lowest number (19) of PCGs, compared to other five mtDNAs.

### Variable Repeat Lengths and Locations Among mtDNAs of *Phaeocystis globosa* Strains

To test our hypothesis that plasticity of repeat regions may be an important driver of *P. globosa* mtDNA diversity in evolution, we constructed mtDNAs of four additional *P. globosa* strains (CNS00069, CNS00087, CNS00262, and CNS00266). The mtDNAs of these strains were still incomplete, suggesting the presence of complex repeat sequences that remain to be resolved. Nevertheless, all genes were successfully identified in these sequences, suggesting that the missing sequences were repeat regions most likely without containing any genes. Aside from the repeat regions, mtDNAs were also relatively compact which was similar to that of strain CNS00066. AT contents of these strains were 66.7-67.2%, which were a little lower than the complete mtDNA of CNS00066.

**FIGURE 6:**
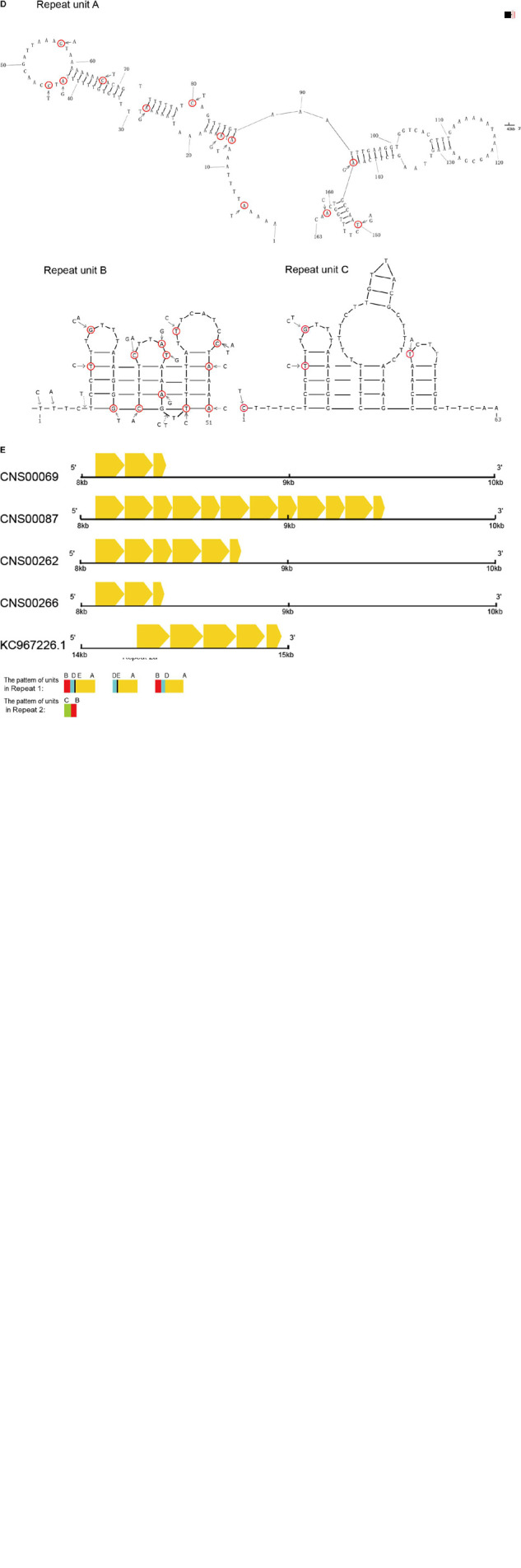
Repeat analysis of *P. globosa.* **(A)** Comparison of the repeat regions position and length among *P. globosa* strains. The black blocks represent the complete repeat regions, and their length was marked under blocks. The gray rectangle showed the end of the scaffold (marked with 1000N in the sequence), and this region is another repeat region which is unobtainable until now. The genome ends in a red vertical line. **(B)** Dot plot similarity matrix of *P. globosa* strain CNS00066 mtDNA against itself. Dots in the nucleotide similarity matrix represent regions of sequence similarity. The main diagonal represents the mtDNA on the *x*-axis matching against its partner of the *y*-axis, Dots adjacent to the main diagonal correspond to repeat DNA and the result indicated two large repeat regions. Dot plots were generated with Dotter under default parameters. **(C)** Repeat structures in *P. globosa* strain CNS00066 mtDNA. Six repeat units (A-F) of different length (13 bp-203 bp) were identified in repeat regions in the strain CNS00066. **(D)** Repeat units A, B, and C exhibit a strong potential to form hairpin secondary structures. **(E)** The repeat unit A showed variable length, sequence similarity and copy numbers among different strains. The yellow arrow boxes represent repeat unit A.

The mtDNAs of all *P. globosa* strains contained two noticeable repeat regions with varying lengths in different strains: repeat 1 and repeat 2 (Figure 6A). The length of complete repeat regions repeat 1 (larger than 250 bp were analyzed here) was rather variable among different *P. globosa* strains, which varied substantially between 334 bp-6.7 kb, with the longest repeat found in the strain CNS00066, and the shortest repeat found in strain CNS00266, suggesting that the repeat regions might be plastic. The gray rectangles in Figure 6A showed the end of the scaffold (represented using a segment of 1000N), and these regions were repeat 2 which are not yet constructed. The still unresolved repeat 2 within all *P. globosa* strains was located at the beginning of two transcription units, while repeat 1 was located at the ending of two transcription units. Within the mtDNA of the *P. antarctica* strain CCMP1374, three repeat regions were found, among which only one complete repeat region was obtained (763 bp in length), and two unfinished repeat regions were located at the ends of two transcription units. The current known repeats length of *Phaeocystis* strains was substantially shorter than the corresponding repeat in strain CNS00066.

The locations of the repeat regions were also variable among different strains. Within the strains CNS00066 and Pg-G(A), two repeat regions were located between block c and block d, block e and block a, respectively. Within strains (CNS00069, CNS00087, CNS00262, and CNS00266), two repeats were between block a and block b, block e and block c, respectively. Gene structure rearrangement was close to repeat regions, such as the block b in strains (CNS00069, CNS00087, CNS00262, and CNS00266) was inverted compared with that of CNS00066. This suggested that rearrangement in mtDNA of *Phaeocystis* may have occurred through repeat-mediated recombination.

### Characterization of Repeat Units in the *P. globosa* Strain CNS00066

Two complex repeat regions were observed in mtDNA of strain CNS00066 (Figure 6B). Six repeat units (A-F) of different length (13 bp-203 bp) were identified in repeat regions in the strain CNS00066 (Figure 6C and Table 3). The number of copies of each repeat unit varied dramatically and the copies of each repeat unit were not identical. For example, repeat unit A (∼163 bp) had 21 copies with sequence identity ranging from 94.4 to 100%; Repeat unit B (∼51 bp) had 252 copies with sequence identity ranging from 63 to 100%; Repeat unit C (∼63 bp) had 48 copies with sequence identity ranging from 92 to 100%; Repeat unit D (34 bp) had 22 copies with sequence identity ranging from 91.1 to 100%; Repeat unit E (13 bp) had 18 copies with sequence identity ranging from 92.3 to 100%; Repeat unit F (∼203 bp) had only two copies with sequence identity of 99.0%. Interestingly, the 5′ 26 bp of the repeat unit C showed high similarity to repeat unit B, suggesting possible evolutionary relationship between these two repeat units.

**TABLE 3:** Repeat units within mtDNA of *P. globosa* strain CNS00066 and their sequence homology.

Repeat unit	Copies number	Sequence length	Sequence matrix length	PID
A	21	161-167 bp	162bp	94.4-100%
B	252	46-54 bp	46bp	63-100%
C	48	63-64 bp	63bp	92.0-100%
D	22	34bp	33bp	91.1-100%
E	18	13bp	13bp	92.3-100%
F	2	203-204 bp	204 bp	99.0%

Repeat region 1 can be divided into two segments repeat 1a and repeat 1b. A large inverted repeat p1 (which consisted of a palindrome sequence A-E-D-B- B-D-E-A) encompassing 573 bp spanning repeat 1a and repeat 1b. Repeat 1 was composed of repeat units A, B, D and E in various arrangements, mainly including B-D-E-A (∼260 bp) repeat units, D-E-A (∼210 bp) repeat units, and B-D-A (∼248 bp) repeat units.

Repeat 2 also consisted of two segments, repeat 2a and repeat 2b, and a large inverted repeat p2 (which is a palindrome arrangement of repeat units B-F-F-B, 588 bp) spanning repeat 2a and repeat 2b. Repeat 2 featured repeat units B, C and F. Maximal 106 consecutive tandem copies of repeat unit B were within repeat 2a. Repeat units B and C showed various arrangements with B-C (114 bp) repeat units being the common arrangement.

The palindromic sequences p1 and p2 which were adjacent to inversion points, exhibiting a strong potential to form stable stem–loop structures (Supplementary Figure 2). The repeat units A, B, and C were also predicted to form stable stem-loop DNA structures (Figure 6D), and repeat unit B was predicted to fold into double-hairpin elements. Variation sites were circled in red. No stem-loop DNA structure was found in repeat units D, E and F.

### Homologous Repeat Sequence Found in *P. globosa* mtDNAs

Because repeat regions were identified in all sequenced mtDNAs of *Phaeocystis* species, we hypothesized that the repeat units identified in different mtDNAs share homology. To test this hypothesis, we analyzed the repeats of mtDNAs of five *P. globosa* strains obtained in this study, in addition to the *P. globosa* strain Pg-G(A) and the *P. antarctica* strain. Results showed that homologous repeat unit A could be identified in all *P. globosa* mtDNAs (Figure 6E), but not in the *P. antarctica* mtDNA.

The repeat unit A showed variable copy numbers, different length and sequence similarities among different strains (Figure 6E). The lengths varied between 55 bp-161 bp. The repeat unit A appeared three times in strain CNS00069, 12 times in strain CNS00087, six times in strain CNS00262, three times in strain CNS00266, and five times in strain Pg-G(A). No homologous sequences were found within *P. antarctica*. The results indicated the repeat unit A may share a common ancestor within *P. globosa*.

## Discussion

The mtDNAs are generally compact with short intergenic regions (Gray et al., 1998; Boore, 1999). However, the sizes of mtDNAs can vary considerably among related species and even between strains of same species, which have been found to be driven by differences in repeat content or the inclusion of introns (Liu et al., 2017). The mtDNAs sequences of two *Ulva pertusa* strains Up1 and Up2 were different by 4.7 kb different in length, which was due to two additional group II introns in two genes (*cox*1 and *cox*2) and tandem duplication mutations in non-coding regions in the larger mtDNA (Liu et al., 2017). A pair of 3.5 kb-long, *cox*1-harboring repeats were found only in two *Nannochloropsis* strains CCMP527 and CCMP537, while intergenic regions of the six mtDNAs were generally conserved (Wei et al., 2013). *Metschnikowia* yeasts mtDNAs vary extensively in size and mainly contributed by different numbers and sizes of introns (Lee et al., 2020).

This study reported the first full-length mtDNA of *P. globosa*, which was 43,585 in size. This large mtDNA size was driven by two large complex repeat regions, whose combined length was almost half of the entire mtDNA size. Indeed, the length of repeat sequences in *P. globosa* was the longest repeat region among all revolved haptophyte mtDNAs. The repeat regions were results of amplification of a few repeat units. The sequences among different repeat unit copies within strain were not identical, and base substitutions and indels existed. Repeat unit could be ubiquitous mutational hotspots. We also identified sequence homology among different repeat regions in the same strains, such as repeat unit B both exist in repeat 1 and repeat 2 in strain CNS00066. This indicated that repeat unit may have been amplified among different repeat regions within mtDNA. The structural features of the repeat regions of *P. globosa* have some similarities compared to other algae such as cryptophyte *Rhodomonas salina* (Hauth et al., 2005) and *Hemiselmis andersenii* (Kim et al., 2008), diatom *Phaeodactylum tricornutum* (Oudot-Le Secq and Green, 2011), and Chlorophyte *Volvox carteri* (Smith and Lee, 2010) which all were composed of several different repeat units, and usually include tandem repeat, reverse repeat and palindromic sequences.

Among *P. globosa* different strains, the length and location of repeat regions, number of repeat units were highly variable, but there is some regularity. For example, repeat unit A was identified in all *P. globosa* strains. It was not found in *P. antarctica*, suggesting that it may be *P. globosa*-specific. The repeat unit A retained obvious sequence similarity among different strains, and this indicated repeat unit A may have a shared origin, existing in the mtDNA of the most recent common ancestor of strains. The sequence similarity of repeat unit A among different strains was often lower than that among different copies within strains, suggesting that the repeat regions acquired mutations in different strains. The fast mutation rates of repeat regions have also been reported in species *Ulva lactuca* (Liu et al., 2020) and *Ulva pertusa* (Liu et al., 2017). Sequence similarity of repeat regions have been reported between related chlorophyte species *Haematococcus lacustris* and *Stephanosphaera pluvialis*, suggesting recent common ancestor of these two species (Zhang et al., 2019; Smith, 2020).

All *P. globosa* strains shared same numbers genes, and these gene sequences were mostly conserved among all *P. globosa* strains, consistent with previous observations that mtDNAs are generally compact (Gray et al., 1998). Genes are encoded on both DNA strands and their orientation suggests the presence of two transcription units within *P. globosa* mtDNAs, e.g., starting from the repeat region 2 and ending in repeat region 1 in both transcription directions in strain CNS00066.

In addition to differences in repeat content among mtDNAs of different strains, genome rearrangement events were also identified. Repeat regions, especially palindromic sequences were adjacent to rearrangement of the mtDNA, suggesting that they may play an important role in genomic rearrangements, transcription and replication, as proposed for other algae including red alga *Chondrus crispus* (Leblanc et al., 1995) and cryptophyte algae *Rhodomonas salina* (Hauth et al., 2005). Microhomologies have also been associated with tandem duplications and structural variation in plant mtDNAs (Xia et al., 2020). Interesting, the palindromic sequence p2 are coincident with a change in the direction of “cumulative GC skew.” Additionally, the palindromic sequence p2 had minimum CG content (Supplementary Figure 3), suggesting that the palindromic sequence p2 may correspond to the origin of replication.

Among the known 18 mtDNAs of haptophyte species, nine were reported as complete sequences, and other nine were reported as partial sequences. Nine full-length mtDNAs all contain repeat regions of various lengths ranging from 1.9 to 20.7 kb (Puerta et al., 2004; Smith and Keeling, 2012; Hovde et al., 2014, 2019; Nishimura et al., 2014; Smith et al., 2014; Hulatt et al., 2020; Wideman et al., 2020). The mtDNA of the *P. globosa* strain CNS00066 had the longest repeat region among all constructed haptophyte mtDNAs. The repeat regions were usually composed of tandem repeat formed by multiple repeat units, such as *Phaeocystis* strains and *Chrysochromulina tobinii* CCMP291, *Pavlova* strains, and *Diacronema viridis* strains. *Chrysochromulina tobinii* CCMP291 has the second longest repeat regions in Haptophyta, which was ∼9.3 kb and consisted of four repeat units ranging from 85 bp to ∼1.5 kb. *Chrysochromulina* sp. NIES-1333 showed a very different mitochondrial structure from other haptophyte strains described above. The *Chrysochromulina* sp. NIES-1333 genome (34,291 bp) has two small intergenic repeats of 1624 bp and 1630 bp, and missing a large, complex repeated sequence array.

Seven of nine partial mtDNAs were obviously truncated in the repeat regions, including those of *Pavlova lutheri* CCMP 1325 and all *Phaeocystis* strains. No obvious repeat regions were found in *Chrysochromulina parva* UW 1161, Haptophyta sp. isolate H2 and *P. ranunculiformis* NIES-3900, and their mtDNAs are partial sequences and have not been successfully assembled. Homologous sequences of the repeat region of *P. globosa* have not been discovered in other haptophyte species. Length of repeat region of different *P. globosa* strains was variable. The repeat length polymorphism maybe used as molecular marker for specific strains.

Phylogenetic analysis using mtDNAs revealed that Rappephyceae was sister to the Pavlovophyceae, while Prymnesiophyceae split from their common ancestor earlier. However, the support was only moderate (Figure 2B), suggesting that the evolutionary relationships among these three classes (Rappephyceae, Pavlovophyceae, and Prymnesiophyceae) might be more complicated. Indeed, our 18S rDNA-based phylogenetic analysis and a recent study suggested that Rappephyceae was sister to the Prymnesiophyceae, while Pavlovophyceae split from their common ancestor earlier (Kawachi et al., 2021). Their exact evolutionary relationship will be resolved with more genomes are sequenced and assembled.

## Conclusion

In this study, we successfully constructed the full-length mtDNA of *P. globosa* for the first time. It was a circular genome with a size of 43,585 bp, encoding 19 PCGs, 25 tRNA genes and two rRNA genes. Surprisingly it was found to contain two large-sized repeat regions of 6.7 kb and ∼14.0 kb in length, respectively. The combined length of these two repeat regions accounted for almost half of the entire mtDNA. The length and the repetitive nature of these two repeat regions made it challenging to construct the complete length of full-length mtDNA of *P. globosa*. Further analysis revealed that the repeat regions consisted of combinations of several of repeat units. To explore the nature of repeat sequences in other *P. globosa* strains, we have also constructed mtDNAs of four additional *P. globosa* strains (one from Beibu Gulf, China and three from Vietnam). Comparative analysis revealed that corresponding repeat regions were readily identified in all strains but with different lengths. A homologous repeat unit A was found to be prevalent among all *P. globosa* strains, but with different numbers of copies and length. In addition to differences in repeats, these mtDNAs also differed in gene structure rearrangement. Another surprising result identified in this project was the plasticity of the repeat regions of the mtDNAs of different *P. globosa* strains. Thus, repeats may be an important driving force of mtDNA evolution. In addition, the mtDNAs of different *P. globosa* strains showed variations, suggesting that the mtDNAs can be used as a super molecular marker for distinguishing different strains.

## Data Availability Statement

The original contributions presented in the study are publicly available. This data can be found here: https://www.ncbi.nlm.nih.gov/nuccore/MW435860,MW435863, MW435865,MW435861,MW435862; https://www.ncbi.nlm.nih. gov/nuccore/MN927483,MN927485,MN927500,MW575291,MW 575290, https://dataview.ncbi.nlm.nih.gov/object/SRR14711855, and http://www.ncbi.nlm.nih.gov/bioproject/732653.

## Author Contributions

NC conceived of the project. HS and YC organized the strain selection, cultivation, DNA preparation, and genome sequencing. HS organized the assembly, annotation, quality control, and analyzed the data with suggestions from others. HS and NC wrote the manuscript with inputs from others. FL revised the manuscript. All authors read and approved the final version of the manuscript.

## Conflict of Interest

The authors declare that the research was conducted in the absence of any commercial or financial relationships that could be construed as a potential conflict of interest.

## Footnotes

1 http://blast.ncbi.nlm.nih.gov/

2 http://ogdraw.mpimp-golm.mpg.de/

3 http://rna.urmc.rochester.edu/RNAstructureWeb/

## Supplementary Material

Supplementary Figure 1 The mitochondrion genomes content and synteny of *P. antarctica* strain CCMP1374 compared to that of strain CNS00066.

Supplementary Figure 2 The partial palindromic (contains the repeat units near loop) sequences of p1 and p2. They exhibit a strong potential to form hairpin secondary structures.

Supplementary Figure 3 GC content and GC skew of *P. globosa* strain CNS00066 mtDNA. GC content (black) and GC skew (green and purple). For GC skew, the center line indicates the average GC skew value for the genome. Green shading above the line denotes GC skew values greater than the genome average, whereas purple shading below the line denotes GC skew values less than the genome average. Major and minor tick marks on the outermost and innermost circles show nucleotide position on the genome in 1 kb increments. The arrow points to loop of the palindromic sequence p2 in repeat region 2.

## References

[B1] Bankevich A. Nurk S. Antipov D. Gurevich A. A. Dvorkin M. Kulikov A. S. (2012). SPAdes: a new genome assembly algorithm and its applications to single-cell sequencing. *J. Comput. Biol.* 19 455–477. 10.1089/cmb.2012.0021 22506599 PMC3342519

[B2] Barson G. Griffiths E. (2016). SeqTools: visual tools for manual analysis of sequence alignments. *BMC Res. Notes* 9:39. 10.1186/s13104-016-1847-3 26801397 PMC4724122

[B3] Bellaousov S. Reuter J. S. Seetin M. G. Mathews D. H. (2013). RNAstructure: web servers for RNA secondary structure prediction and analysis. *Nucleic Acids Res.* 41 W471–W474. 10.1093/nar/gkt290 23620284 PMC3692136

[B4] Benson G. (1999). Tandem repeats finder: a program to analyze DNA sequences. *Nucleic Acids Res.* 27 573–580. 10.1093/nar/27.2.573 9862982 PMC148217

[B5] Blauw A. N. Los F. J. Huisman J. Peperzak L. (2010). Nuisance foam events and *Phaeocystis globosa* blooms in Dutch coastal waters analyzed with fuzzy logic. *J. Mar. Syst.* 83 115–126. 10.1016/j.jmarsys.2010.05.003

[B6] Boore J. L. (1999). Animal mitochondrial genomes. *Nucleic Acids Res.* 27 1767–1780. 10.1093/nar/27.8.1767 10101183 PMC148383

[B7] Cao X. Yu Z. Qiu L. (2017). Field experiment and emergent application of modified clays for *Phaeocystis globosa* blooms mitigation (in Chinese, with English abstract). *Oceanol. Limnol. Sin.* 48 753–759.

[B8] Capella-Gutierrez S. Silla-Martinez J. M. Gabaldon T. (2009). trimAl: a tool for automated alignment trimming in large-scale phylogenetic analyses. *Bioinformatics* 25 1972–1973. 10.1093/bioinformatics/btp348 19505945 PMC2712344

[B9] Chen Y. Q. Wang N. Zhang P. Zhou H. Qu L. H. (2002). Molecular evidence identifies bloom-forming Phaeocystis (Prymnesiophyta) from coastal waters of southeast China as *Phaeocystis globosa*. *Biochem. Syst. Ecol.* 30 15–22. 10.1016/S0305-1978(01)00054-0

[B10] Darling A. E. Mau B. Perna N. T. (2010). progressiveMauve: multiple genome alignment with gene gain, loss and rearrangement. *PLoS One* 5:e11147. 10.1371/journal.pone.0011147 20593022 PMC2892488

[B11] Glez-Pena D. Gomez-Blanco D. Reboiro-Jato M. Fdez-Riverola F. Posada D. (2010). ALTER: program-oriented conversion of DNA and protein alignments. *Nucleic Acids Res.* 38 W14–W18. 10.1093/nar/gkq321 20439312 PMC2896128

[B12] Gray M. W. Lang B. F. Cedergren R. Golding G. B. Lemieux C. Sankoff D. (1998). Genome structure and gene content in protist mitochondrial DNAs. *Nucleic Acids Res.* 26 865–878. 10.1093/nar/26.4.865 9461442 PMC147373

[B13] Greiner S. Lehwark P. Bock R. (2019). OrganellarGenomeDRAW (OGDRAW) version 1.3.1: expanded toolkit for the graphical visualization of organellar genomes. *Nucleic Acids Res.* 47 W59–W64. 10.1093/nar/gkz238 30949694 PMC6602502

[B14] Guillard R. R. L. Hargreaves P. E. (1993). Stichochrysis-immobilis is a diatom, not a chrysophyte. *Phycologia* 32 234–236. 10.2216/i0031-8884-33-1-66b.1

[B15] Hai D. N. Lam N. N. Dippner J. W. (2010). Development of *Phaeocystis globosa* blooms in the upwelling waters of the South Central coast of Viet Nam. *J. Mar. Syst.* 83 253–261. 10.1016/j.jmarsys.2010.04.015

[B16] Hall T. A. (1999). BioEdit: a user-friendly biological sequence alignment editor and analysis program for windows 95/98/NT. *Nucleic Acids Symp. Ser.* 41 95–98.

[B17] Hauth A. M. Maier U. G. Lang B. F. Burger G. (2005). The Rhodomonas salina mitochondrial genome: bacteria-like operons, compact gene arrangement and complex repeat region. *Nucleic Acids Res.* 33 4433–4442. 10.1093/nar/gki757 16085754 PMC1183108

[B18] Hovde B. T. Deodato C. R. Andersen R. A. Starkenburg S. R. Barlow S. B. Cattolico R. A. (2019). Chrysochromulina: Genomic assessment and taxonomic diagnosis of the type species for an oleaginous algal clade. *Algal Res. Biomass Biofuels Bioprod.* 37 307–319. 10.1016/j.algal.2018.11.023

[B19] Hovde B. T. Starkenburg S. R. Hunsperger H. M. Mercer L. D. Deodato C. R. Jha R. K. (2014). The mitochondfrial and chloroplast genomes of the haptophyte *Chrysochromulina tobin* contain unique repeat structures and gene profiles. *BMC Genomics* 15:604. 10.1186/1471-2164-15-604 25034814 PMC4226036

[B20] Hu Z. X. Deng Y. Y. Tang Y. Z. (2019). Scanning and transmission electron microscopy observation on morphology and ultrastructure of *Phaoeycstis globosa* from Beibu Gulf, China (in Chinese, with English abstract). *Oceanol. Limnol. Sin.* 50 621–629.

[B21] Hulatt C. J. Wijffels R. H. Viswanath K. Posewitz M. C. (2020). The complete mitogenome and plastome of the haptophyte *Pavlova lutheri* NIVA-4/92. *Mitochondrial DNA B Resour.* 5 2748–2749. 10.1080/23802359.2020.1788436 33457933 PMC7782304

[B22] Jin J. J. Yu W. B. Yang J. B. Song Y. dePamphilis C. W. Yi T. S. (2020). GetOrganelle: a fast and versatile toolkit for accurate de novo assembly of organelle genomes. *Genome Biol.* 21:241. 10.1186/s13059-020-02154-5 32912315 PMC7488116

[B23] Johnson M. Zaretskaya I. Raytselis Y. Merezhuk Y. McGinnis S. Madden T. L. (2008). NCBIBLAST: a better web interface. *Nucleic Acids Res.* 36 W5–W9. 10.1093/nar/gkn201 18440982 PMC2447716

[B24] Katoh K. Standley D. M. (2013). MAFFT multiple sequence alignment software version 7: improvements in performance and usability. *Mol. Biol. Evol.* 30 772–780. 10.1093/molbev/mst010 23329690 PMC3603318

[B25] Kawachi M. Nakayama T. Kayama M. Nomura M. Miyashita H. Bojo O. (2021). Rappemonads are haptophyte phytoplankton. *Curr. Biol.* 31. 10.1016/j.cub.2021.03.012 [Epub ahead of print]. 33773100

[B26] Kim E. Lane C. E. Curtis B. A. Kozera C. Bowman S. Archibald J. M. (2008). Complete sequence and analysis of the mitochondrial genome of *Hemiselmis andersenii* CCMP644 (Cryptophyceae). *BMC Genomics* 9:215. 10.1186/1471-2164-9-215 18474103 PMC2397417

[B27] Krzywinski M. Schein J. Birol I. Connors J. Gascoyne R. Horsman D. (2009). Circos: an information aesthetic for comparative genomics. *Genome Res.* 19, 1639–1645. 10.1101/gr.092759.109 19541911 PMC2752132

[B28] Kumar S. Stecher G. Tamura K. (2016). MEGA7: Molecular evolutionary genetics analysis version 7.0 for bigger datasets. *Mol. Biol. Evol.* 33 1870–1874. 10.1093/molbev/msw054 27004904 PMC8210823

[B29] Lagesen K. Hallin P. Rodland E. A. Staerfeldt H. H. Rognes T. Ussery D. W. (2007). RNAmmer: consistent and rapid annotation of ribosomal RNA genes. *Nucleic Acids Res.* 35 3100–3108. 10.1093/nar/gkm160 17452365 PMC1888812

[B30] Leblanc C. Boyen C. Richard O. Bonnard G. Grienenberger J. M. Kloareg B. (1995). Complete Sequence of the Mitochondrial-DNA of the Rhodophyte *Chondrus-crispus* (Gigartinales) - Gene Content and Genome Organization. *J. Mol. Biol.* 250 484–495. 10.1006/jmbi.1995.0392 7616569

[B31] Lee D. K. Hsiang T. Lachance M. A. Smith D. R. (2020). The strange mitochondrial genomes of Metschnikowia yeasts. *Curr. Biol.* 30 R800–R801. 10.1016/j.cub.2020.05.075 32693070

[B32] Li H. Bloom J. M. Farjoun Y. Fleharty M. Gauthier L. Neale B. (2018). A synthetic-diploid benchmark for accurate variant-calling evaluation. *Nat. Methods* 15 595–597. 10.1038/s41592-018-0054-7 30013044 PMC6341484

[B33] Li H. Durbin R. (2009). Fast and accurate short read alignment with Burrows-Wheeler transform. *Bioinformatics* 25 1754–1760. 10.1093/bioinformatics/btp324 19451168 PMC2705234

[B34] Li H. Handsaker B. Wysoker A. Fennell T. Ruan J. Homer N. (2009). The Sequence Alignment/Map format and SAMtools. *Bioinformatics* 25 2078–2079. 10.1093/bioinformatics/btp352 19505943 PMC2723002

[B35] Liu F. Melton J. T. Bi Y. P. (2017). Mitochondrial genomes of the green macroalga *Ulva pertusa* (Ulvophyceae, Chlorophyta): novel insights into the evolution of mitogenomes in the Ulvophyceae. *J. Phycol.* 53 1010–1019. 10.1111/jpy.12561 28677163

[B36] Liu M. M. Liu F. Chen N. S. Melton J. T. Luo M. B. (2020). Mitochondrial genomes and phylogenomic analysis of *Ulva lactuca* Linnaeus (Ulvophyceae, Chlorophyta). *Mitochondrial DNA Part B Resour.* 5 1638–1639. 10.1080/23802359.2020.1745712

[B37] Lowe T. M. Chan P. P. (2016). tRNAscan-SE On-line: integrating search and context for analysis of transfer RNA genes. *Nucleic Acids Res.* 44 W54–W57. 10.1093/nar/gkw413 27174935 PMC4987944

[B38] Madhupratap M. Sawant S. Gauns M. (2000). A first report on a bloom of the marine prymnesiophycean, *Phaeocystis globosa* from the Arabian Sea. *Oceanol. Acta* 23 83–90. 10.1016/S0399-1784(00)00109-2

[B39] Nishimura Y. Kamikawa R. Hashimoto T. Inagaki Y. (2014). An intronic open reading frame was released from one of group II introns in the mitochondrial genome of the haptophyte *Chrysochromulina* sp. NIES-1333. *Mob. Genet. Elements* 4:e29384. 10.4161/mge.29384 25054084 PMC4091101

[B40] Oudot-Le Secq M. P. Green B. R. (2011). Complex repeat structures and novel features in the mitochondrial genomes of the diatoms *Phaeodactylum tricornutum* and *Thalassiosira pseudonana*. *Gene* 476 20–26. 10.1016/j.gene.2011.02.001 21320580

[B41] Puerta M. V. S. Bachvaroff T. R. Delwiche C. F. (2004). The complete mitochondrial genome sequence of the haptophyte *Emiliania huxleyi* and its relation to heterokonts. *DNA Res.* 11 1–10. 10.1093/dnares/11.1.1 15141941

[B42] Qi Y. Z. Chen J. F. Wang Z. H. Xu N. Wang Y. Shen P. P. (2004). Some observations on harmful algal bloom (HAB) events along the coast of Guangdong, southern China in 1998. *Hydrobiologia* 512 209–214. 10.1023/B:HYDR.0000020329.06666.8c

[B43] Rombel I. T. Sykes K. F. Rayner S. Johnston S. A. (2002). ORF-FINDER: a vector for high-throughput gene identification. *Gene* 282 33–41. 10.1016/S0378-1119(01)00819-8 11814675

[B44] Rousseau V. Chretiennot-Dinet M. J. Jacobsen A. Verity P. Whipple S. (2007). The life cycle of Phaeocystis: state of knowledge and presumptive role in ecology. *Biogeochemistry* 83 29–47. 10.1007/s10533-007-9085-3

[B45] Rousseau V. Lantoine F. Rodriguez F. LeGall F. Chretiennot-Dinet M. J. Lancelot C. (2013). Characterization of *Phaeocystis globosa* (Prymnesiophyceae), the blooming species in the Southern North Sea. *J. Sea Res.* 76 105–113. 10.1016/j.seares.2012.07.011

[B46] Schoemann V. Becquevort S. Stefels J. Rousseau W. Lancelot C. (2005). Phaeocystis blooms in the global ocean and their controlling mechanisms: a review. *J. Sea Res.* 53 43–66. 10.1016/j.seares.2004.01.008

[B47] Seuront L. Vincent D. Mitchell J. G. (2006). Biologically induced modification of seawater viscosity in the Eastern English Channel during a *Phaeocystis globosa* spring bloom. *J. Mar. Syst.* 61 118–133. 10.1016/j.jmarsys.2005.04.010

[B48] Shen P. P. Qi Y. Z. (2021). Research progress on species diversity and distribution of the genus phaeocystis. *Oceanol. Limnol. Sin.* 52 1–15.

[B49] Shen P. P. Wang Y. Qi Y. Z. Me L. C. Lv S. H. Hodgkiss I. J. (2000). Growth characteristics and life cycle of *Phaeocystis globosa* Scherffel (in Chinese, with English abstract). *Acta Hydrobiol. Sin.* 24 635–643.

[B50] Smith D. R. (2020). Common repeat elements in the mitochondrial and plastid genomes of green algae. *Front. Genet.* 11:465. 10.3389/fgene.2020.00465 32477407 PMC7235400

[B51] Smith D. R. Arrigo K. R. Alderkamp A. C. Allen A. E. (2014). Massive difference in synonymous substitution rates among mitochondrial, plastid, and nuclear genes of *Phaeocystis* algae. *Mol. Phylogenet. Evol.* 71 36–40. 10.1016/j.ympev.2013.10.018 24216019

[B52] Smith D. R. Keeling P. J. (2012). Twenty-fold difference in evolutionary rates between the mitochondrial and plastid genomes of species with secondary red plastids. *J. Eukaryot. Microbiol.* 59 181–184. 10.1111/j.1550-7408.2011.00601.x 22236077

[B53] Smith D. R. Lee R. W. (2010). Low nucleotide diversity for the expanded organelle and nuclear genomes of *Volvox carteri* supports the mutational-hazard hypothesis. *Mol. Biol. Evol.* 27 2244–2256. 10.1093/molbev/msq110 20430860

[B54] Song H. Y. Liu F. Li Z. L. Xu Q. Chen Y. Yu Z. M. (2020). Development of a high-resolution molecular marker for tracking *Phaeocystis globosa* genetic diversity through comparative analysis of chloroplast genomes. *Harmful Algae* 99:101911. 10.1016/j.hal.2020.101911 33218437

[B55] Thorvaldsdottir H. Robinson J. T. Mesirov J. P. (2013). Integrative Genomics Viewer (IGV): high-performance genomics data visualization and exploration. *Brief. Bioinform.* 14 178–192. 10.1093/bib/bbs017 22517427 PMC3603213

[B56] Tillich M. Lehwark P. Pellizzer T. Ulbricht-Jones E. S. Fischer A. Bock R. (2017). GeSeq - versatile and accurate annotation of organelle genomes. *Nucleic Acids Res.* 45 W6–W11. 10.1093/nar/gkx391 28486635 PMC5570176

[B57] Trifinopoulos J. Nguyen L. T. von Haeseler A. Minh B. Q. (2016). W-IQ-TREE: a fast online phylogenetic tool for maximum likelihood analysis. *Nucleic Acids Res.* 44 W232–W235. 10.1093/nar/gkw256 27084950 PMC4987875

[B58] Turmel M. Lemieux C. Burger G. Lang B. F. Otis C. Plante I. (1999). The complete mitochondrial DNA sequences of *Nephroselmis olivacea* and *Pedinomonas minor*: two radically different evolutionary patterns within green algae. *Plant Cell* 11 1717–1729. 10.1105/tpc.11.9.1717 10488238 PMC144307

[B59] Wei L. Xin Y. Wang D. M. Jing X. Y. Zhou Q. Su X. Q. (2013). Nannochloropsis plastid and mitochondrial phylogenomes reveal organelle diversification mechanism and intragenus phylotyping strategy in microalgae. *BMC Genomics* 14:534. 10.1186/1471-2164-14-534 23915326 PMC3750441

[B60] Wei W. Jiang T. J. (2005). Studies on the toxicity of two strains *Phaeocystis globosa* scherffeto *Artemia sinica* (in Chinese, with English abstract). *Ecol. Sci.* 24 38–41.

[B61] Wideman J. G. Monier A. Rodriguez-Martinez R. Leonard G. Cook E. Poirier C. (2020). Unexpected mitochondrial genome diversity revealed by targeted single-cell genomics of heterotrophic flagellated protists. *Nat. Microbiol.* 5 154–165. 10.1038/s41564-019-0605-4 31768028

[B62] Xia H. H. Zhao W. Shi Y. Wang X. R. Wang B. S. (2020). Microhomologies are associated with tandem duplications and structural variation in plant mitochondrial genomes. *Genome Biol. Evol.* 12 1965–1974. 10.1093/gbe/evaa172 32790831 PMC7643612

[B63] Xiao C. L. Chen Y. Xie S. Q. Chen K. N. Wang Y. Han Y. (2017). MECAT: fast mapping, error correction, and de novo assembly for single-molecule sequencing reads. *Nat. Methods* 14 1072–1074. 10.1038/Nmeth.4432 28945707

[B64] Zhang Q. C. Niu Z. Wang J. X. Liu C. Kong F. Z. Hu X. K. (2021). Development of high-resolution chloroplast markers for intraspecific phylogeographic studies of *Phaeocystis globosa*. *J. Oceanol. Limnol.* 39 508–524. 10.1007/s00343-020-9304-5

[B65] Zhang X. Bauman N. Brown R. Richardson T. H. Akella S. Hann E. (2019). The mitochondrial and chloroplast genomes of the green alga Haematococcus are made up of nearly identical repetitive sequences. *Curr. Biol.* 29 R736–R737. 10.1016/j.cub.2019.06.040 31386847

