# Supplementary figures and images for: Large Differences in the Haptophyte Phaeocystis globosa Mitochondrial Genomes Driven by Repeat Amplifications
Source: Front Microbiol. 2021 Jul 2;12:676447. doi: 10.3389/fmicb.2021.676447 (PMC8283788; doi:10.3389/fmicb.2021.676447)

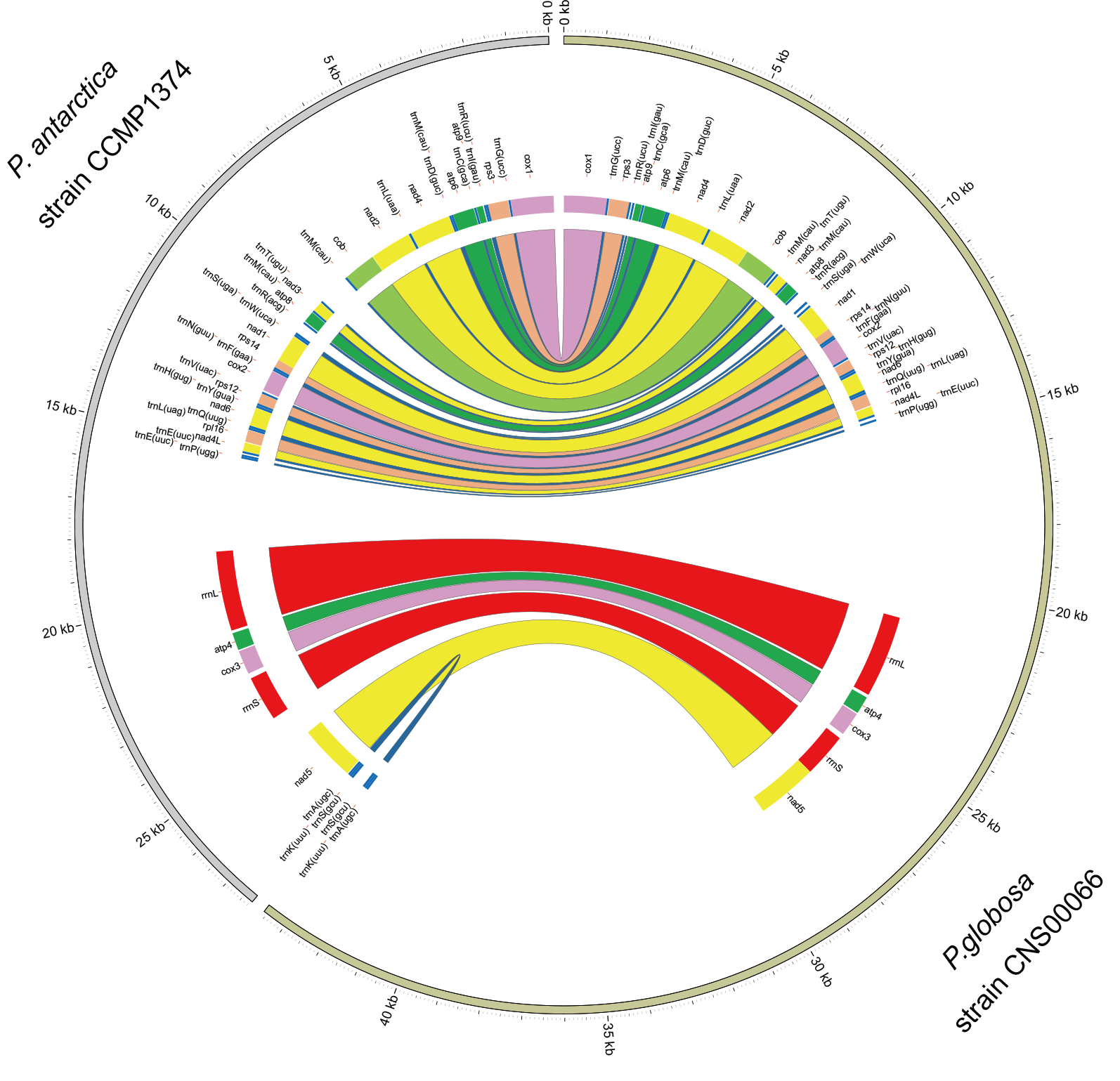

Supplement: Supplementary Figure 1 — The mitochondrion genomes content and synteny of P. antarctica strain CCMP1374 compared to that of strain CNS00066. [file Image_1.TIF]

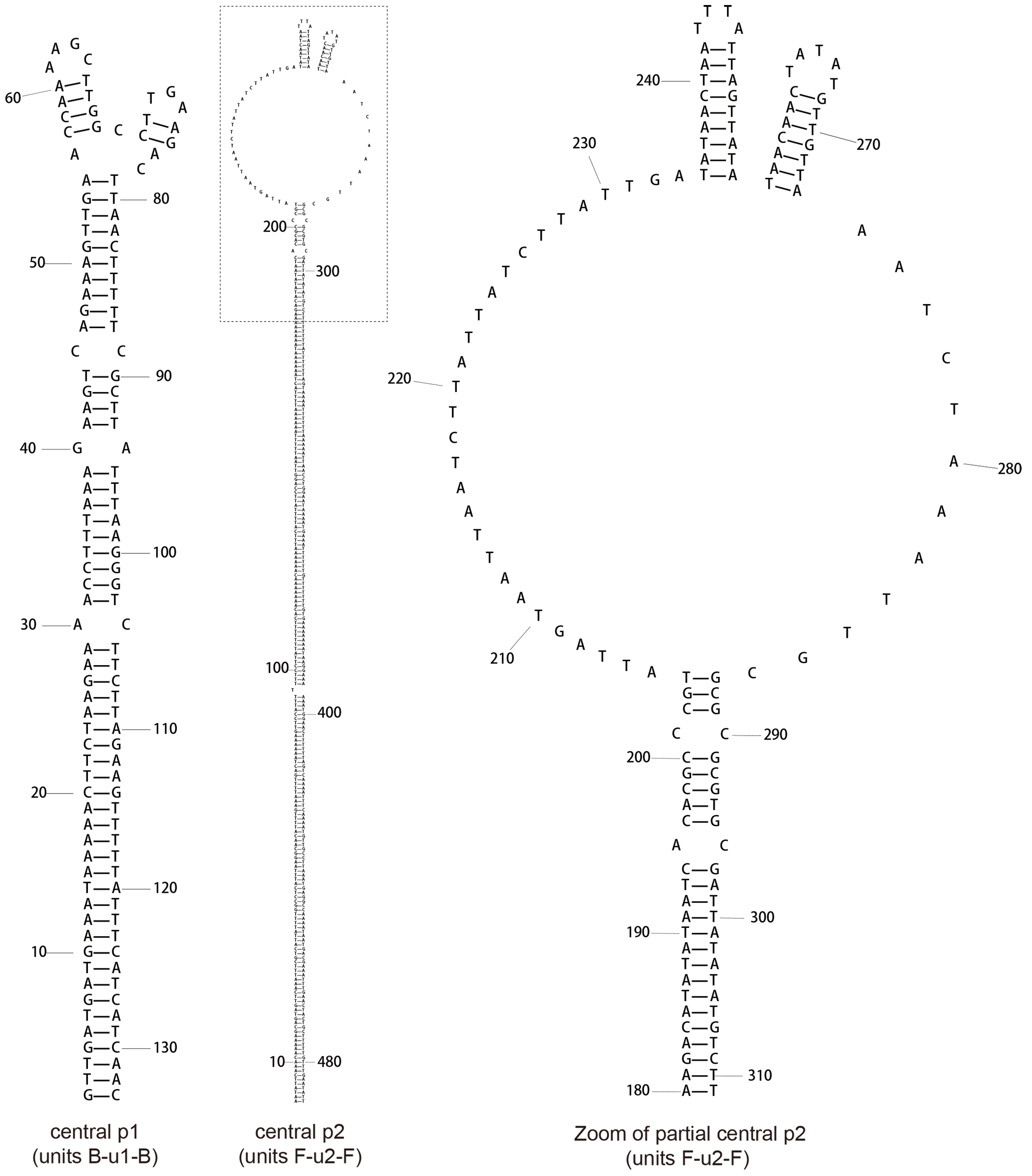

Supplement: Supplementary Figure 2 — The partial palindromic (contains the repeat units near loop) sequences of p1 and p2. They exhibit a strong potential to form hairpin secondary structures. [file Image_2.TIF]

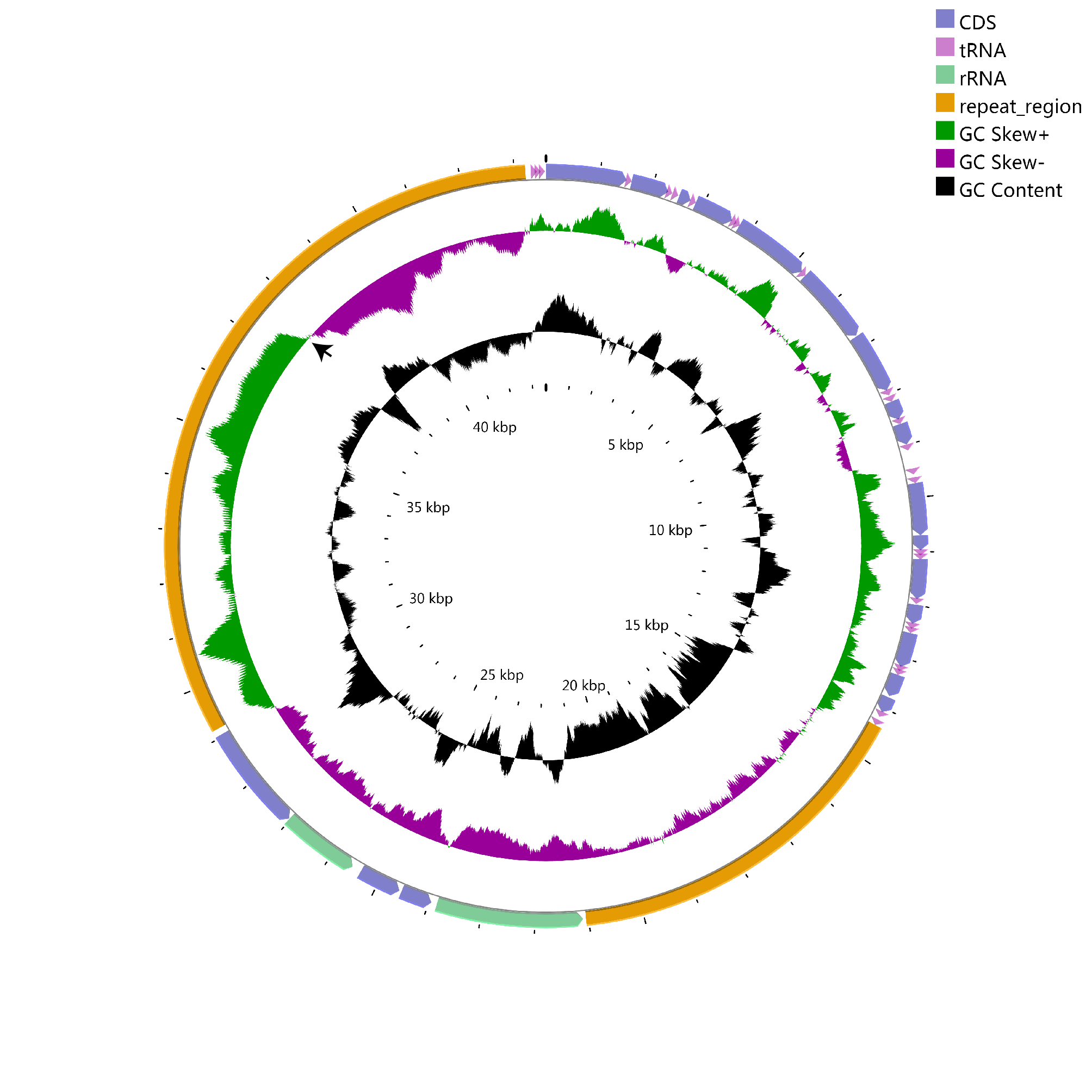

Supplement: Supplementary Figure 3 — GC content and GC skew of P. globosa strain CNS00066 mtDNA. GC content (black) and GC skew (green and purple). For GC skew, the center line indicates the average GC skew value for the genome. Green shading above the line denotes GC skew values greater than the genome average, whereas purple shading below the line denotes GC skew values less than the genome average. Major and minor tick marks on the outermost and innermost circles show nucleotide position on the genome in 1 kb increments. The arrow points to loop of the palindromic sequence p2 in repeat region 2. [file Image_3.TIF]
